# Challenges in the Diagnosis of *Taenia solium* Cysticercosis and Taeniosis in Medical and Veterinary Settings in Selected Regions of Tanzania: A Cross-Sectional Study

**DOI:** 10.1155/2022/7472051

**Published:** 2022-06-30

**Authors:** Fredy Mlowe, Esron Karimuribo, Ernatus Mkupasi, Ayubu Churi, Antony D. Nyerere, Veronika Schmidt, Helena Ngowi, Andrea S. Winkler, James Mlangwa

**Affiliations:** ^1^Ileje District Council, Ileje, Songwe, Tanzania; ^2^Department of Veterinary Medicine and Public Health, College of Veterinary Medicine and Biomedical Sciences, Sokoine University of Agriculture, Morogoro, Tanzania; ^3^Centre for Information and Communication Technology (CICT), Sokoine University of Agriculture, Morogoro, Tanzania; ^4^Center for Global Health, Department of Neurology, Technical University of Munich, Munich, Germany; ^5^Centre for Global Health, Institute of Health and Society, University of Oslo, Oslo, Norway

## Abstract

**Background:**

*Taenia solium* (neuro) cysticercosis/taeniosis (TSCT) is a zoonotic disease complex. There is a perceived inefficient diagnosis of infections by either form, the adult pork tapeworm (taeniosis) and the larval stage of it (cysticercosis), in low-income settings, including Tanzania. This study aimed at identifying potential gaps around TSCT diagnosis and knowledge of primary healthcare providers (officers in charge (OICs) of primary healthcare facilities (PHFs)) and veterinarians (meat inspectors (MIs)) on various aspects of TSCT disease complex and addressing effective disease control in Tanzania. *Methodology*. A cross-sectional study was conducted between January and April 2020 in Manyara, Dodoma, Ruvuma, Iringa, and Arusha regions in Babati, Mbulu, Kongwa, Mbinga, and Nyasa districts. We interviewed 152 OICs of PHFs and 108 MIs using a structured questionnaire and 33 medical and veterinary officers from level I healthcare facilities and district livestock offices, respectively, from selected study districts to the respective ministerial level using key informant interviews.

**Results:**

Quantitative data revealed inadequate microscopic diagnostic facilities (54.6%) and personnel (100%) for taeniosis diagnosis in PHFs (*n* = 152). Approximately 81.2% of MIs compared with only 42.1% of OICs of PHFs scored above average regarding *T. solium* cysticerci knowledge. Nevertheless, 61.2% of OICs of PHFs compared with only 42.6% of MIs scored above average regarding the adult *T. solium* tapeworm knowledge. Qualitative data revealed inadequate availability of advanced diagnostic facilities (neuroimaging) and trained personnel for specific diagnosis of TSCT with a focus on neurocysticercosis (NCC) in secondary and tertiary healthcare facilities. Inadequately number of qualified MIs, slaughter slabs, and resource facilitation challenged porcine cysticercosis diagnosis.

**Conclusion:**

It is concluded that diagnostic capacity and knowledge of OICs of PHFs and MIs regarding TSCT are insufficient in both medical and veterinary sectors. A One Health approach should be adopted to improve TSCT diagnostic capacity and practitioners' knowledge in both medical and veterinary sectors.

## 1. Introduction

*Taenia solium* is a zoonotic parasite that causes *T. solium* (neuro) cysticercosis/taeniosis (TSCT) that is transmitted between humans and pigs and between humans themselves [1]. The life cycle of the parasite involves humans, who are the definitive hosts, and pigs, who are intermediate hosts [1]. The parasite life cycle maintenance between humans and pigs is attributed to poor sanitation, unhygienic practices in food preparation, inadequate hygienic measures of the slaughter house/slab personnel, improper handling of infected pig carcasses, whereby *T. solium* cysticercosis-positive pig carcass are not properly buried or incinerated, and poor pig husbandry practices in which pigs are left to scavenge on human faeces and eat feeds and drink water contaminated by *T. solium*-infective eggs [2].

Human infection by *T. solium* tapeworm causes taeniosis, which occurs through consumption of improperly cooked pork infected by *T. solium* larvae. Taeniosis in humans is characterized by potential abdominal discomforts including diarrhoea and abdominal cramps. Some individuals remain entirely asymptomatic [3]. The disease has also been incriminated to be responsible for chronic anaemia [4] and weight loss in some patients [5]. When humans ingest food contaminated by infective *T. solium* eggs, they develop cysticercosis, which is the presence of cysticerci in human tissues [6]. Improperly washed hands by the tapeworm carrier or using unclean water, which is more likely to be contaminated by *T. solium*-infective eggs, to wash hands, fruits, or vegetables is highly associated with increased likelihood of getting infected with *T. solium* cysticercosis [7].

Presence of *T. solium* cysticerci in the central nervous system (CNS), referred to as neurocysticercosis (NCC), is the most common clinically important life-threatening health condition caused by *T. solium* tapeworms [8]. However, invasion of other vital organs such as heart by the tapeworm cysticerci can also lead to life-threatening health condition [9]. NCC may cause neurological manifestations such as epileptic seizures and/or chronic progressive headache, among others. The intensity of these neurological signs/symptoms as well as their frequency depends on the size, number, location, and inflammatory stage of the cyst in the brain [10]. Pigs get infected by *T. solium* cysticerci through scavenging on human faeces and eating feeds contaminated by infective *T. solium* eggs [6]. Pigs with *T. solium* cysticerci in the brain have been shown to experience neurological signs similar to those expressed by humans with NCC [11].

In Tanzania, taeniosis prevalence of 2.3% and 5.2% has been estimated using Kato-Katz and copro-antigen enzyme-linked immunosorbent assay (ELISA), respectively [12]. On the other hand, based on antigen ELISA, human cysticercosis prevalence of 16.3% (of which 19% had NCC suggestive lesions under CT scan) in Mbulu [13] and 16.7% in Mbozi [7] has been reported. Studies have shown that human NCC accounts for 29.0% [14] to 32.3% of all epileptic cases in TSCT-endemic countries [15]. Globally, NCC and porcine cysticercosis (PCC) have been associated with considerable economic burdens in both medical and veterinary sectors [16–18]. High prevalence of PCC reported in various parts of the country is a reflection of potential economic losses [12, 19, 20].

A number of diagnostic tests for TSCT disease complex are in use to date, including stool microscopy (for taeniosis), serology, and molecular tests (for taeniosis and (neuro)cysticercosis) and radioimaging (for NCC) [8]. In Tanzania, *Taenia* species proglottids are occasionally expulsed and seen in faeces, towel, and sometimes undergarments by tapeworm carriers [21]. Thus, the history of expulsion of *Taenia* species has been used as a tool in taeniosis diagnosis [22]. However, especially in *T. solium*, the excretion of proglottids and eggs is very intermittent and passive (in contrast to the active migration of *T. saginata* and *T asiatica* proglottids). This irregular excretion makes visual and microscopic *T. solium* taeniasis diagnosis unreliable. Thus, the absence of proglottids or eggs in the stool does not always indicate the absence of taeniosis [22–24]. Moreover, microscopy has low specificity, ranging from 22.5% to 56.0% [24], as eggs of *T. solium* and those of *T. saginata* look similar under the light microscope and thus almost always only genus-level diagnosis has been possible [25]. For the accurate differentiation of the three tapeworm species—*T. solium*, *T. saginata*, and *T. asiatica*—polymerase chain reaction (PCR) techniques with or without subsequent sequencing are required [26]. However, these techniques are expensive, require specialized training of personnel, and are therefore not available in most areas.

Immunological methods such as copro-antigen enzyme-linked immunosorbent assay (CoAg-ELISA) using stool samples and enzyme-linked immunoelectrotransfer blot (EITB) using serum are used to diagnose taeniasis [27]. CoAg-ELISA identifies specific *T. solium* tapeworm antigens in human stool with a specificity and sensitivity of about 100% and 96%, respectively, being reported [28]. The test is clinically useful in monitoring treatment outcome following antigen disappearance few weeks after commencing the treatment [29]. Antigen ELISA has been used to diagnose human cysticercosis from serum samples [29]. Antibody-ELISA on the other hand is used to identify antibodies produced by the host immune system in serum samples [30]. Gabriel et al. [31] reported the B158/B60 monoclonal-based antibody-ELISA specificity and sensitivity of 100.0% and 84.0%, respectively, in diagnosing *T. solium* NCC. Nevertheless, despite high sensitivity, the test lacks specificity and is unable to differentiate between active and previous cysticercosis infection [32]. With this regard, *T. solium* LLGP-based EITB has been developed and is reported to have 98.0% and 100% sensitivity and specificity, respectively [33]. The test is capable of detecting antibodies to any of the seven lentil-lectin-bound glycoproteins of *T. solium* cyst extract and is a “gold standard” serodiagnostic tool in supporting the diagnosis of *T. solium* NCC [34].

Imaging techniques such as computed tomography (CT) scanners and magnetic resonance imaging (MRI) are considered confirmatory tests in diagnosing NCC [35]. However, not only are they expensive and inadequately available in resource-limited countries, but also they are challenged by inability to differentiate NCC lesions from other neurological pathological conditions [35]. The fact that no single diagnostic test can confirm NCC diagnosis subjected Del Brutto et al. [36] into proposing a number of criteria to be taken into account to come up with a definitive or probable diagnosis of *T. solium* NCC [36]. The criteria took into account, among others, the strengths of various diagnostic methods and were broadly classified into absolute criteria and those pertaining to neuroimaging results and clinical manifestations as well as risk of exposure. Each of these was further classified into histological confirmation of the parasite, evidence of subretinal cyst, and demonstration of the scolex in the cyst (for absolute criteria); major, confirmative, and minor (for neuroimaging criteria); and major and minor (for clinical/exposure criteria), the latter including, among others, detection of specific anticysticercal antibodies or cysticercal antigens by well standardized immunodiagnostic tests or cysticercosis outside the CNS (major), evidence of contact with a household *T. solium* carrier (major), suggestive clinical manifestation, and residency in endemic countries (minor) [36].

PCC diagnosis employs tongue palpation and postmortem meat inspection in addition to molecular and serodiagnostic techniques used in human taeniosis and cysticercosis/NCC diagnosis. Tongue palpation for PCC diagnosis lies on identification of palpable viable cysticerci under the tongue [37]. The technique has a sensitivity of up to 21% and specificity of up to 100.0% [38]. Meat inspection, which is widely and commonly used in diagnosing PCC, lies on identification of cysticerci in striated muscles and the brain [39]. The method is reported to have lower sensitivity in diagnosing the disease in lightly infected pigs [40]. There are various serological techniques available for diagnosing PCC. Antibody-ELISA is reported to have high sensitivity but poor specificity with inability to distinguish between active and previous exposure. A sandwich-antigen detection ELISA on the other hand is reported to have sensitivity and specificity ranges of 82.7% and 86.3% [41] to 82.9% and 96.8% [42], respectively. The whole crude *T. solium* cysticercus antigen (WCA)-based EITB assay is reported to have high sensitivity and specificity [42]. However, its sensitivity and specificity in naturally PCC-infected pig diagnosis have been questionable [43]. This may be attributed by the fact that the test is incapable of diagnosing *T. solium* PCC in naturally lightly infected pigs [44].

It is generally evident that diagnosis of TSCT and NCC is challenged by unavailability of diagnostic tools with high sensitivity and specificity to accurately diagnose the disease complex in both hosts [45]. Despite the diagnostic tools' performance challenges, in LMICs in particular, the diagnosis of TSCT is also challenged by inadequate accessibility and affordability of these tools [46]. Strategies towards control/elimination of TSCT rely on efficient and coordinated One Health surveillance and monitoring systems that enable reliable estimation of the disease magnitude in both pigs and humans [41]. Tanzania, like other LMICs, could likely be facing challenges in diagnosing TSCT in pigs and humans, though little is known in this aspect as to how big the challenge is. This study aimed at assessing the diagnostic capacity of medical and veterinary health facilities and practitioners' knowledge (OICs of PHFs and MIs) towards TSCT diagnosis in Tanzania to identify gaps in the diagnosis of this One Health disease complex in both medical and veterinary sectors, which, if effectively addressed, will contribute to One Health TSCT surveillance and successful control/elimination strategies of the disease complex.

## 2. Materials and Methods

### 2.1. Description of Study Areas

**Figure 1:**
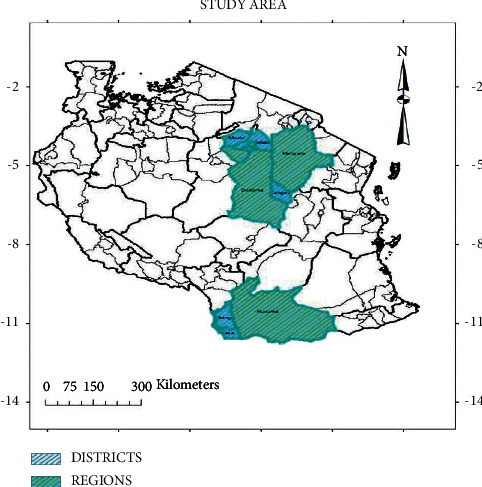
Study districts to assess challenges and opportunities in the diagnosis of *T solium* taeniosis/cysticercosis in Tanzania.

The study was conducted in five purposively selected regions of Tanzania, namely, Ruvuma, Iringa (southern Tanzania), Dodoma (central Tanzania), Manyara, and Arusha (northern Tanzania). The selected regions were to have either districts in which the study was to be conducted, based on the reported prevalence of *T. solium* PCC of 12% or more by previous studies [12], or presence of zonal veterinary investigation centres (ZVCs) and Tanzania Veterinary Laboratory Agency (TVLA) to which samples for possible PCC laboratory testing could be submitted. It involved five districts, namely, Babati and Mbulu (Manyara region), Kongwa (Dodoma region), Mbinga, and Nyasa districts (Ruvuma region) (Figure 1).

In Tanzania, primary (dispensaries and healthcare centres) and level I healthcare facilities are qualified to be equipped with basic laboratory equipment including microscopes. They would be expected to be able to diagnose and manage taeniosis though not necessarily at species level. This is because the highly sensitive diagnostic tools with high specificity such as PCR are lacking at this level. Such healthcare facilities are not qualified to be equipped with advanced diagnostic equipment capable of diagnosing *T. solium* cysticercosis or NCC in humans [47]. On the other hand, level II and III healthcare facilities, which include regional and zonal referral hospitals, are higher level healthcare facilities qualified to have specialized diagnostic equipment and physicians. They are the referral hospitals for cases from level I healthcare facilities and are qualified to have specialized personnel and radiologic diagnostic facilities such as CT and MRI (for level III healthcare facilities) scanners. These healthcare facilities (II and III) were expected to be able to diagnose *T. solium* NCC in addition to taeniosis cases.

In this study, the diagnosis of TSCT was conceptualized in two aspects: the diagnosis of the adult *T. solium* tapeworm in humans and the diagnosis of the larval stage of the tapeworm (cysticerci) in both human and pigs. With the level of diagnostic tools at PHFs, which are widely distributed all around the country than level I, II, and III healthcare facilities, only taeniosis diagnosis was expected to be possible at this healthcare level. Hence, it was important to assess the presence of functioning laboratories in PHFs capable of doing at least stool analysis and diagnose helminthosis including taeniosis. In addition, considering the complex life cycle of the parasite, it was important to assess the general knowledge of the personnel (OICs) of PHFs on both forms of the parasite. With this regard, the prepared questionnaire aimed at assessing both aspects to OsIC of PHFs with questions assessing the knowledge on *T. solium* tapeworm subjecting him/her into describing the tapeworm, its larval stage (cysticerci) morphology, and the dynamics behind human infection by both forms of the parasite. Assessment of TSCT diagnosis by MIs was featured towards diagnosis of the larval stage of the parasite in pigs (PCC) as this is the only form/stage of the parasite in pigs. Therefore, the efficiency of TSCT diagnosis in this aspect was measured by the presence of pig slaughter houses/slabs in the respective wards/villages in the study districts, presence of adequate trained/skilled MIs, and the presence of facilitation on day-to-day meat inspection activities particularly in terms of means of transport. Explanation on the decision and management of the diagnosed PCC cases by MIs was incurred to understand the contribution of PCC-positive cases on TSCT disease maintenance in endemic areas. Likewise, the knowledge of MIs on the parasite was considered in addition to the diagnosis of PCC, and therefore a questionnaire was prepared to assess both aspects by MIs. Questions assessing MIs' knowledge on *T. solium* tapeworm subjected them into describing the tapeworm and its larval stage (cysticerci) morphology and the dynamics behind human and pig infection by both forms of the parasite. Knowledge regarding TSCT by the respondents of the structured questionnaire was reflected by their level of knowledge score to each of the individual aspect assessed (that is, awareness, general description of the adult and larval stage of the parasite morphology, and the dynamics behind human and pig infection by both forms of the parasite). NCC diagnosis could only be possible at levels II and III. Therefore, key informant interview guide questions were specifically prepared to capture information on the available capacity to diagnose this form of the parasite in humans in addition to diagnosis of taeniosis and PCC as per one-to-one key informants' perceptions.

### 2.2. Study Design

This was a cross-sectional study conducted from January to April 2020 involving quantitative and qualitative methods of data collection for triangulation purposes. Structured questionnaires were used to collect quantitative data from OICs of PHFs and MIs, while one-to-one in-depth interview with key informants was used to collect qualitative data.

Quantitative data were collected from MIs and medical health respondents who were OICs of PHFs. Qualitative data were collected from both medical and veterinary officers working from level I healthcare facilities (public or private level I hospital) and the DLFDD respectively to the national level. The medical respondents targeted for qualitative data were the medical doctors (MDs) (or whoever who would respond on his/her behalf in case of his/her absence), whereas the respondents targeted from the veterinary sector were the district veterinary officers (DVOs) or district livestock and fisheries officers (DLFOs) in the absence of a DVO at district level and veterinary doctor or livestock officer (in the absence of veterinary doctor) at any higher level beyond district level.

### 2.3. Sample Size and Participants Selection

A total of 297 respondents (167 from OICs of PHFs and 130 from MIs in wards or villages) were selected to be administered with structured questionnaire interviews from a total target population of 478 using the formula *n*=*N*/(1+*N*(*e*)^2^), where *n* = study sample size, *N* = study population size, and *e* = level of precision: *n*=192/(1+192(0.05)^2^) = 130 for MIs and *n*=286/(1+286(0.05)^2^) = 167 for OICs of PHFs.

The number of OICs of PHFs and MIs included in the study from each district was determined based on the probability proportional to size sampling approach. The number of livestock and agricultural field officers doing meat inspection in wards and villages in the veterinary sector were recruited into the district sample size based on the proportion each of the two strata contributed to the total number of extension officers in each district. Both MIs and OICs of PHFs were randomly selected from the list of MIs and PHFs in the district provided by the district veterinarian or livestock officer and district medical officer, respectively.

A total of 33 respondents from medical and veterinary sectors were selected using the purposive sample selection approach from level I healthcare facilities (mainly district hospitals) and DLFDD, respectively, to the ministerial level to be interviewed using one-to-one key informants interview basing on the criteria that they are experts in the field to be sure that they provide expertise opinion/perception regarding a research topic.

### 2.4. Data Collection

#### 2.4.1. Quantitative Data

Quantitative data were collected from OICs of PHFs and MIs using Afyadata application [48] in which structured questionnaires were digitalized on a tablet and a smart phone. Two questionnaires, one for OICs of PHFs and another for MIs, were pretested on 10 respondents (5 OICs of PHFs and 5 MIs), each with a respective questionnaire and adjustments were made to questions that needed to be edited. All respondents gave their verbal consents to participate following the researcher's briefing of the research purpose and importance. The structured questionnaires were administered to the respondents (OICs of PHFs and MIs) by reading and explaining the questions and available responses to the respondents in Swahili language. A respondent had to choose response(s) that he/she thought they best described the phenomenon in question from the responses read to him/her by the interviewer (Supplement (S1)).

#### 2.4.2. Qualitative Data

One-to-one in-depth interviews with the key informants was conducted using an interview guide with questions which were prearranged to focus on the known diagnostic approaches to specific disease conditions as stipulated in the World Health Organization/International Organisation for Animal Health/World Food and Agricultural Organization (WHO/OIE/FAO) standard guidelines for specific diseases diagnosis and surveillance [49]. We interviewed a total of 16 and 17 respondents from medical and livestock sectors, respectively, who were purposively chosen to capture their perception on taeniosis, NCC/epilepsy, and PCC diagnostic capacity in their working healthcare facilities and those below/above them. Before the assessment of diagnostic capacity availability of any of the TSCT disease conditions, the researcher ensured that the respondent was referring to the disease condition in question by introducing the topic to the respondent before the actual interview. Also respondent's responses to follow-up questions further highlighted whether or not the respondent was referring to the disease in question. When the researcher noted unawareness regarding the condition, no further questions were asked. The interviews lasted for 35 minutes on average and were recorded using an audio recorder following verbal consent by the respondent.

### 2.5. Data Analysis

#### 2.5.1. Quantitative Data Analysis

Quantitative data were analyzed in SPSS. Frequencies and proportions were computed to determine the number and percentage of responses towards a variable of interest. Association between respondent's variable of interest and correct responses were analyzed using the chi-square test. *P*-values less than 0.05 (*P* < 0.05) were considered statistically significant. An open-ended question asked to 108 MIs requiring them to briefly explain how they go about doing *T. solium* PCC risk-based meat inspection was analyzed under qualitative subheading since responses to this question were more of qualitative than quantitative data.

The scores obtained in this analysis and those for the open-ended question, which was asked to MIs only (analyzed under the qualitative data analysis), were converted into percentages and then categorized into four as follows: excellent knowledge (those who scored 70% or more), good knowledge (between 60% and 69%), average knowledge (those who scored between 50 and 59%), and poor knowledge (those who scored below 50% of the total scores).

#### 2.5.2. Qualitative Data Analysis

Qualitative data collected using an audio recorder were transcribed and translated into English in a Microsoft Word document. The collected data were analyzed using ATLAS.ti qualitative data analysis software. Content analysis was used to analyze the transcripts, and it involved both deductive and inductive approaches, which involved extensive reading of each transcript to grasp concepts discussed by the respondents. A concept was considered to be a theme (important) idea if it reached a cutoff point of at least 50% (at least 50% of the respondents mentioned the concept).

An open-ended question to MIs was analyzed as follows: A total of 108 transcripts were made and imported into ATLAS.ti software for important ideas to be coded and analyzed. Based on pork inspection guideline [50], a total of 8 scores were created as follows: each respondent mentioning antemortem pig inspection as one of the pork inspection procedures got 1 score, and an additional score was given for each procedure mentioned on postmortem pork carcass inspection, that is, visualization, palpation, and incision (3 scores in total). In addition, for each cysticercus commonly known predilection site mentioned, that is, tongue, masseter, and heart, one (1) score each was given (making a total of 3 scores). Lastly, for any additional organ(s) mentioned by the respondent, one extra score was given. This made up a total of 8 scores.

## 3. Results

### 3.1. Quantitative Study Results

We interviewed a total of 260 participants out of the targeted 297, of which 186 (71.5%) were males; 152 were OICs of PHFs, of which 95 (62.5%) were males; and 108 were MIs, of which 91 (84.3%) were males. Pig slaughter slabs were admitted not to be present by 90.7% of the MIs with all MIs (100%) admitting the absence of transport facilitation in their daily meat inspection activities. A total of only 20 pig slaughter slabs were reported to be present. In addition, there were only 52 (33.6%) PHFs capable of doing stool analysis.  Table 1 provides the study population and distribution.

#### 3.1.1. Diagnosis of *T. solium* Taeniosis

Out of the 152 OICs of PHFs interviewed, 101 (66.4%) admitted the absence of a laboratory with the necessary equipment (including a microscope) to do stool analysis for helminthosis/taeniosis diagnosis. As such, 87 (57.2%) of respondents admitted to rely on patients' clinical history, mainly of reported expulsion of tapeworm pieces in stool to diagnose taeniosis, while the rest opted for referring them to nearby health centres or hospitals for stool analysis.

#### 3.1.2. Knowledge of Officers in Charge of Primary Healthcare Facilities regarding *T. solium* Taeniosis and (Neuro)cysticercosis

Out of 152 OICs of PHFs interviewed, 133 (87.5%) and 72 (47.5%) admitted to be aware of *T. solium* tapeworm and *T. solium* cysticerci in pigs, respectively.  Table 2 provides the knowledge of OICs of PHFs regarding *T. solium* tapeworm and *T. solium* cysticerci.

The level of knowledge regarding *T. solium* tapeworms and *T. solium* cysticerci among OICs of PHFs is given in Table 3.

Respondents with clinical medical health profession (CMHP) training were significantly more aware than the other medical professionals (OMPs), which included enrolled nurses 17 (50%), registered nurses 6 (17.6%), medical attendants 6 (17.6%), midwives 2 (5.9%), assistant nurses 2 (5.9%), and laboratory assistant 1 (2.9%), regarding *T. solium* tapeworm (*P*=0.009, *n* = 112, 73.7%) and NCC (*P*=0.005, *n* = 56, 36.8%). In addition, more respondents with CMHP correctly described NCC compared with those with OMPs (*P*=0.017, *n* = 34, 22.4%). There was no significant difference in medical respondents with different working experience who were aware about *T. solium* cysticerci (*P*=0.005). The study also found no significant difference regarding knowledge about other various parameters on *T. solium* biology and epidemiology (transmission dynamics) in relation to differences in professional background and working experience of OICs of PHFs. More male medical respondents were aware about *T. solium* tapeworms than female (*P* < 0.001, *n* = 90, 59.2%), whereas there was no significant difference in awareness about *T. solium* cysticerci between sex. In addition, a large proportion of male respondents correctly described the mode of human infection by *T. solium* cysticerci compared with female respondents (*P*=0.032, *n* = 72, 47.4%).

#### 3.1.3. Knowledge of Meat Inspectors on *T. solium* Taeniosis and Porcine Cysticercosis

The knowledge of MIs regarding various aspects of TSCT is given in Table 4.

MIs' knowledge scores on different aspects of TSCT disease complex and porcine cysticercosis risk-based pork carcass inspection are given in Table 5.

A large proportion of MIs with professional background other than animal health, which included diploma in crop production 23 (39.0%), diploma in agro-mechanisation 13 (22.0%), diploma in range management and tsetse control 13 (22.0%), bachelor of science in range management 3 (5.1%), bachelor of science in animal science and production 2 (3.4%), and bachelor of science in agronomy 1 (1.7%), appeared to be aware about *T. solium* adult worms than those of animal health profession (AHP) (*P*=0.009, *n* = 47, 43.5%) despite absence of significant differences in knowledge on other various parameters regarding *T. solium* biology between professional background or working experience of MIs. In addition, a significantly large proportion of male MIs correctly described the risk factors for *T. solium* parasite life cycle maintenance than female MIs (*P*=0.042, *n* = 283, 87.1%). Mbinga districts had the largest proportion of MIs who correctly described the risk factors for *T. solium* tapeworm life cycle maintenance (*P*=0.001, *n* = 83, 25.5%). This was followed by Kongwa, Babati, Mbulu, and Nyasa. Regional wise, Ruvuma had a significantly larger proportion of MIs with correct description of *T. solium* tapeworm risk factors for life cycle maintenance than other regions (*P*=0.001, *n* = 131, 40.3%). Furthermore, the Ruvuma region had a higher proportion of MIs who correctly described *T. solium* cysticerci than other regions (*P*=0.007, *n* = 99, 41.3%).

### 3.2. Qualitative Study Results

Table 6 provides the distribution by geographical and administrative levels of 33 respondents.

#### 3.2.1. *T. solium* Taeniosis Diagnosis in Primary Healthcare Facilities as Perceived by Medical Health Respondents

A total of 12 quotations were captured with regard to presence of a qualified laboratory technician for taeniosis diagnosis in the study areas. Out of these two admitted availability, three admitted inadequate availability and seven admitted complete unavailability of competent laboratory technician in most of the PHFs as evidenced by the following quotation: (“the challenge for taeniosis diagnosis is the deficiency in laboratory and technicians in health facilities especially in dispensaries”—Male, District Medical Officer). In addition, out of the 11 quotations regarding availability of diagnostic facilities for taeniosis, five indicated that they were completely unavailable, while six indicated that diagnostic facilities were inadequately available in primary health facilities and district hospitals (“but another challenge I think is the fact that there is absence of helminthosis testing ability, laboratories are few,” female, Acting District Medical Officer). Furthermore, three out of four quotations admitted that the diagnostic personnel, which included clinical officers, assistant medical officers, and medical officers, were not sufficiently available in primary health facilities (“If you tell someone to diagnose *Taenia* in the diagnosis or taeniosis while he is not laboratory expert and he doesn't have laboratory, they are just medical attendants and others are just nurses it is difficult,” male, District Medical Officer). Six out of eight quotations admitted that level I healthcare facilities had facilities and personnel capable of diagnosing taeniosis (“….yes at least there, we have the laboratory personnel, but these hospitals which are two in our council, are religion owned, which are Litembo and Ruanda. These have got personnel and are capable of diagnosing helminthosis, we don't have government hospital in our council yet,” male, District Medical Officer).

#### 3.2.2. Human Neurocysticercosis Diagnosis in Secondary Healthcare Facilities as Perceived by Medical Health Respondents

From 14 medical respondents who were interviewed, 10 quotations admitted that diagnostic facilities for NCC were not available in their health facilities and in most of their respective referral hospitals (“here we don't have laboratory investigation for diagnosing whether this is normal epilepsy or this is epilepsy due to NCC,” male, District Hospital Medical Officer). A total of three out of four quotations relating to personnel capable of diagnosing NCC admitted that physicians specialized in the central nervous system health were not available in their healthcare facilities (“Aaah, it is not that easy and I would still say doctors should be sensitized on that matter,… most of cases are treated symptomatically … but if they don't improve they refer them to higher referral hospitals with experts with advanced diagnostic equipment …, so even though worms are within top ten conditions but to think of worms in relation to epilepsy is very rare,” male, District Medical Officer).

#### 3.2.3. Human Neurocysticercosis Diagnosis in Tertiary Healthcare Facilities as Perceived by Medical Health Respondents

Six out of nine quotations admitted that diagnostic facilities for human NCC were not present in their respective referral hospitals (“No, it is not easy, you know, neurocysticercosis is something in advanced stage, so it needs advanced technology, for example, you may need things like a CT scanner, MRI, which we do not have them here, and I think throughout this zone, we do not have the CT scanner, so most likely, we will depend on symptoms …,” male, District Medical Officer). Three out of five quotations relating to personnel capable of diagnosing NCC admitted that physicians specialized in the central nervous system health were not present in most of the healthcare facilities (“…I have been here for a while now, but I have never seen anybody reporting neurocysticercosis in his report. It is possibly there, but people might be under diagnosing it, people may be having epilepsy and the like, but they cannot tell exactly what has caused it,” male, Regional Medical Doctor).

#### 3.2.4. Porcine Cysticercosis Diagnosis as Perceived by Livestock Sector Respondents

Four out of seven quotations admitted that there were no official pig slaughter slabs or slaughter houses in their localities (“the pig owners will slaughter and call meat inspector because we do not have pig slaughter house,” male, District Livestock and Fisheries Officer). Ten out of 12 quotations admitted that MIs were not adequate (“aah, they cannot be enough, I have 25 wards may be… at least if I could manage to have one animal health extension officer it could be much better but as it is now they are not enough,” male, District Veterinary Officer). In addition, eight out of 15 quotations admitted that some of the MIs were not trained for the job (“in my district, in slaughter houses, it is animal health extension officers, but in villages where we have only slaughter slabs I have assigned extension officers with other professional backgrounds like agricultural extension officers,” male, Acting District Livestock and Fisheries Officer), and 12 out of 14 quotations admitted that MIs were not facilitated, meaning that they were not provided with necessary working tools and the means of transport to reach various locations to inspect meat (“aah, to tell you the truth, not only them but also us, there is no any facilitation as of now, in the past, we used to be facilitated somehow,” female, District Veterinary Officer). Furthermore, three out of five quotations from TVLA respondents admitted that porcine cysticercosis was largely diagnosed visually at postmortem meat inspection (“but in most cases, extension officers know porcine cysticercosis in endemic areas, the diagnosis using postmortem procedure is known, so after diagnosing, they do not take further steps in most cases, what is left is how to control it,” male, Officer in Charge of TVLA).

## 4. Discussion

### 4.1. Overview

To the best of our knowledge, this is the first study conducted in Tanzania to assess the TSCT diagnostic capacities in health and livestock sectors concurrently. The use of both quantitative and qualitative data collection approaches provided room for triangulation of some responses. Key informant interviews not only complemented questionnaire responses but also provided additional information on the research topic. Most of the respondents were males due to the fact that more males than females are enrolled in medical and veterinary colleges in Tanzania. Nevertheless, a good number of females were included in the study. Less than the targeted number of respondents was interviewed in this study due to inability to access some rural areas as a result of badly destructed weather roads due to heavy rains during the data collection period. Also the COVID-19 outbreak and its mitigation recommendations limited our access to some of the targeted respondents. This study revealed many challenges that limited effective diagnosis of *T. solium* infections at various levels of the medical and veterinary health facilities, making it difficult to accurately quantify the magnitude of *T. solium* infections. Specific diagnostic capacity issues at each health facility level are discussed below.

### 4.2. Diagnosis of *T. solium* Taeniosis

Our study found that only 33.8% and 77.6% of the PHFs in the study districts had laboratories and OICs with clinical health professional background, respectively. This finding reflects reduction in the efficiency of PHFs, which are widely distributed throughout the country (at least one health centre in each ward) in diagnosing common parasitic conditions including taeniosis. It is therefore difficult to capture the taeniosis prevalence at PHFs, which have countrywide coverage. These are the first healthcare provision facilities most patients will seek health consultation in LMICs but are often insufficiently facilitated to provide the expected healthcare services [51].

Level I health facilities on the other hand were in a position to diagnose most helminthic diseases (including taeniosis, though mostly at genus level), among others, as was reflected by the qualitative part of our study. However, the accessibility of district (and other privately owned level I) hospitals by the resource-limited communities is hindered by financial constraints in most LMICs [52]. In addition, patients' decision to deliberately access or adhere to a referral from a PHF to a level I health facility is challenged by a characteristic negligence of most patients on health challenges, which they perceive to be minor [53]. This could also account for difficulty in capturing taeniosis burden in the country despite the presence of level I health facilities in almost all districts in Tanzania with undoubtable capacity to diagnose helminthosis because patients will hardly deliberately access them. It calls for a need for continuous health education in communities in LMICs with an emphasis on the importance of constantly checking their health status and adhering to health advices provided by their healthcare personnel. Different from our study, a study conducted in India indicated the fact that *T. solium* taeniosis was among the neglected diseases as it was demonstrated by the absence of the need for active identification and treatment of *T. solium* taeniosis by the medical personnel than personnel and diagnostic facility insufficiency that hindered efficient diagnosis of the disease [54].

### 4.3. Diagnosis of Human Neurocysticercosis

The study revealed the insufficient availability of CT and MRI scanners in tertiary hospitals (regional and zonal referral hospitals). This is in line with the WHO report on *T. solium* NCC diagnosis challenges in LMICs whereby unavailability of advanced diagnostic equipment was mentioned among the contributing factors for inefficient NCC diagnosis [46]. Also, a study conducted in Peru, though not specifically on TSCT, indicated that limited access to diagnostic facilities for important debilitating diseases including NCC by most of patients hindered efficient diagnosis of the disease [55]. This altogether reflects the difficulty with which confirmatory diagnosis of NCC or epilepsy-related cases can be in most of the secondary and tertiary hospitals. Moreover, limited availability of NCC diagnostic facilities coupled with high diagnostic services and travelling costs hinders the accessibility of NCC diagnostic services by resource-limited patients [56]. As such, what is currently known about the NCC disease burden is likely to be less than the actual disease prevalence in the community. As a result, any efforts and resources put in place to control or eliminate the disease in the country are likely to be underestimated.

### 4.4. Diagnosis of Porcine Cysticercosis

Inadequate animal health trained MIs, pig slaughter houses/slabs, and the lack of facilitation on the day-to-day meat inspection activities hindered efficient diagnosis of *T. solium* PCC. Adequate availability of slaughter slabs in rural settings where PCC is endemic is important as far as *T. solium* PCC diagnosis is concerned than it could be with laboratory tests, which are expensive and impractical in these settings where PCC can be diagnosed even by naked eyes. This is because slaughter slabs provide the catchment areas to capture PCC burden as pig owners/pork businessmen bring their pigs for slaughter and in a way get inspected and necessary decisions are made to pig carcasses that are found positive for *T. solium* PCC. The inadequate number of pig slaughter slabs in the study districts revealed in our study reflects high chances that a good number of pigs were slaughtered at farmers' homes. Coupled with inadequate number of MIs who were inadequately facilitated to move around to provide meat inspection services, the chance that most of home-slaughtered pigs were consumed uninspected is high. This increases the chances of community exposure to infected pork, taeniosis, and subsequent NCC infection [57]. Similar challenges were revealed by Lwelamira [58] and Levy et al. [59] from Tanzania and Kenya, respectively, whereby pigs were home slaughtered and lately inspected or not inspected at all due to the absence of slaughter slabs and inadequate MIs.

While it may be true that *T. solium* PCC is easily identified in pork carcasses by experienced MIs as was perceived by livestock laboratory experts, low sensitivity of pork carcass inspection procedures to detect cysticerci in lightly infected pigs may represent a loophole for perpetuating *T. solium* infection [40]. This is even more worrying as most of the interviewed livestock sector respondents stated that MIs were not trained adequately. In addition, inadequate knowledge of the public on the potential of PCC to causing taeniosis and subsequently NCC and risk factors for its transmission, including inadequate cooking of pork [60], is likely to potentiate TSCT prevalence in endemic areas.

The health risks posed by the proportions that opted for conditional passing of the perceived lightly infected pork (Table 4) should not be underestimated. This is because there is a narrow chance that consumers would cook the infected pork to recommended temperature to kill the cysticerci [60]. In addition, this is contrary to the provision of the Animal Diseases Regulation of 2007 of Tanzania that demands total condemnation, involving burning of the *T. solium* PCC-positive carcass regardless of the number of cysticerci [61].

Fortunately, in our study, most of the predilection sites for *T. solium* cysticerci in pig carcasses [50] were correctly recognized by MIs (Table 4). This reflects some degree of knowledge and the possibility of diagnosing the disease by a significant proportion of respondents when these sites are actually targeted.

However, the chances of encouraging black market with *T. solium* PCC-positive carcasses diagnosed at meat inspection contributing to a significant portion of PCC-positive carcasses along the pork value chain with the feeling that they are lightly infected being among the excuses should not be underestimated [62]. Also, owing to inadequate facilitation of MIs in their daily meat inspection activities, the possibility of getting tempted and engage in illegal business and negotiation with *T. solium* PCC-positive carcass owners is high [62]. This has a potential of increasing the number of *T. solium* PCC-positive carcasses along the pork value chain and, in a way, increasing the risk of getting infected with PCC and developing taeniosis and subsequent NCC to a significant proportion of the community.

### 4.5. Knowledge of Officers in Charge of Primary Healthcare Facilities on *T. solium* Tapeworm Biology

The majority of medical respondents (87%) were found to be knowledgeable about the *T. solium* adult tapeworm. Working in the *T. solium*-endemic areas may have exposed them to frequent encounters with this tapeworm and thus contributed to improve and maintain their knowledge. However, given the level of health facilities they are working, they are not expected to encounter the larval stage of the parasite. This may have accounted for their inadequate knowledge (only 47% of respondents) regarding the larval stage of the parasite. Limited knowledge of medical respondents on the means of human infection by *T. solium* cysticerci may be due to the fact that *T. solium* was one of the neglected parasites (more than 60% did not give the correct answer) in medical health training [63]. The level of knowledge regarding *T. solium* tapeworm of OICs of PHFs in our study is almost similar to the baseline knowledge on the parasite of the medical health participants of a study conducted in Mbeya that was aimed at assessing efficiency of a *T. solium* health education tool [64]. The reason behind the similarity could partly be explained by the fact that the participants of the two studies were from the same medical profession with different experiences but added advantages of both being working in areas which are taeniosis/(neuro)cysticercosis-endemic. The significant difference in awareness of *T. solium* tapeworm, NCC, and correct definition of NCC among OICs of PHFs with different professional background might be explained by the possibility that they were exposed differently in their medical health training according to their professional courses.

### 4.6. Knowledge of Meat Inspectors regarding *T. solium* Tapeworm Biology

The lower number of MIs (43%) with knowledge regarding *T. solium* tapeworm could be due to the fact that their daily meat inspection and animal health service provision activities do not expose them to the adult stage of the parasite for them to practically know the parasite. This is supported by the fact that the same MIs were knowledgeable about *T. solium* cysticerci whereby 81% of them scored 50% and above regarding the knowledge of the larval stage of the parasite, which they frequently encounter in their daily postmortem pig carcass inspection. Considerable knowledge regarding risk factors for *T. solium* tapeworm life cycle maintenance demonstrated by a large proportion of MIs (65%) explains the potential of MIs in the control/elimination of this important zoonotic parasite in endemic areas. However, a low proportion of MIs (36%) were knowledgeable about *T. solium* porcine cysticercosis risk-based pork carcass inspection. This fact exposes the served community to high risk of getting infected with *T. solium* taeniosis and subsequently NCC as the chance for humans consuming a positive PCC pig carcass is higher. Different from our study, a study conducted in Mbeya aiming at assessing the efficiency of the *T. solium* taeniosis/cysticercosis health education tool had shown higher knowledge of veterinary professional participants at the baseline on porcine cysticercosis diagnosis (48%) than MIs in our study (36.1%) [64]. This could partly be explained by the fact that our study had most of MIs with professional background other than animal health and most of them had graduated some years back without refresher course or in-service training on meat inspection. This is different from participants of the previous study whereby most of them had animal health profession at different levels (diploma to bachelor degrees) and some participants were in their last years of their training with fresh memories and skills.

Male MIs seem to have significantly a higher level of knowledge than female MIs because throughout the study areas there was more male than female MIs. This resulted in recruiting more males than females into the study sample size. As a result, correct responses seem to reflect the number of respondents in each gender than differences in other factors. Likewise, different levels of knowledge among MIs from different districts and regions could be due to differences in the number of participants who participated in the study. This is shown by the fact that those with a higher number of participants had many correct responses than those with fewer participants.

### 4.7. Comparison of the Knowledge of Officers in Charge of Primary Healthcare Facilities and Meat Inspectors

There was a significantly smaller proportion of OICs of PHFs with knowledge regarding *T. solium* cysticerci than that of MIs (47% vs. 99%), which could be explained by the fact that with the level of their healthcare facilities, OICs of PHFs are less exposed to the larval stage of the parasite than MIs who through their daily meat inspection activities are frequently encountering the larval stage of the parasite during meat inspection. Taeniosis being one of the marginalized health problems in medical training could also explain this difference [63].

On the other hand, a significantly smaller proportion of MIs with knowledge about *T. solium* adult worms than OICs of PHFs (*P* < 0.001) is explained by the fact that their daily meat inspection activities do not expose them to the adult stage of the parasite. This is different from OICs of PHFs whose daily routine activities are likely to expose them to adult tapeworm cases from their visiting patients. However, a considerable knowledge on the *T. solium* tapeworm and *T. solium* cysticerci has been shown despite differences in knowledge regarding the two stages of the parasite between and within OICs of PHFs and MIs.

This study is limited by the fact that it excluded laboratory personnel who were not OICs of PHFs, and we therefore did not capture their knowledge and opinions particularly on taeniosis diagnosis. However, given the lower number of PHFs with laboratories, their responses would not significantly affect our findings. Inclusion of respondents with different levels of expertise, in qualitative key informant interviews, working at different levels of healthcare facilities served to mitigate this challenge.

## 5. Conclusion

*T. solium* taeniosis, NCC, and PCC diagnoses remain to be a challenge in both the medical and veterinary sectors. The two forms of the disease in human beings bring about differences in diagnostic hurdles. While taeniosis diagnostic shortcomings were at the level of PHFs, NCC diagnostic challenges were mostly at level II and III healthcare facilities, which were meant to be supported by specialized physicians and diagnostic facilities. Nevertheless, most of them were lacking diagnostic neuroimaging, which is crucial in the diagnosis of *T. solium* NCC. PHFs have a potential of serving large rural communities, which in most cases are at higher risk of taeniosis and subsequent NCC infection. They need to be capacitated with diagnostic facilities and laboratory technicians to effectively serve *T. solium* taeniosis surveillance, which, in turn, will help predicting NCC-vulnerable population countrywide. PCC diagnosis challenges were more on unskilled MIs with inadequate facilitation mostly in terms of mobility. As we struggle to control and eliminate the health and socioeconomic devastating disease complex, there is a need to explore more on the best ways to engage the community to achieve this. It should include, among others, exploring their role in ensuring that only PCC-free pork enters the food chain and how this will reduce the impact of a black market that may be contributed by the absence of MIs or deliberate passing of PCC-positive carcasses by unfaithful or reckless MIs. In addition, in-service training to improve knowledge of both medical and veterinary service providers should be considered. This should be coupled with improved infrastructure and technical and financial support for effective diagnosis and control of TSCT in a true One Health approach.

## Figures and Tables

**Table 1:** Geographical and professional characteristics of respondents.

Variable	Number (%) of respondents	
	OICs of PHFs	MIs
Region		
Manyara	53 (34.4)	38 (34.5)
Dodoma	36 (23.4)	26 (24.5)
Ruvuma	63 (42.2)	44 (40.9)

District		
Babati	30 (19.7)	22 (20.3)
Mbulu	23 (15.1)	16 (14.8)
Kongwa	36 (23.6)	26 (24.1)
Mbinga	42 (27.6)	28 (25.9)
Nyasa	21 (13.8)	16 (14.8)

Type of healthcare facility		
Dispensary	131 (86.2)	NA
Health centres	21 (13.8)	NA

Clinical medical health against other profession		
OICs with clinical medical health background	118 (77.6)	NA
OICs with nonclinical medical health background^*∗*^	34 (22.4)	NA

Animal health profession against other profession		
Animal health	NA	44 (40.7)
Other background^*∗∗*^	NA	64 (59.3)

^*∗*^Nurses, midwives, medical attendants, and laboratory technicians. ^*∗∗*^Agricultural science (bachelor and diploma), agronomist (bachelor), animal scientists (bachelor and diploma), and agro-mechanisation (diploma). OICs, officers in charge.

**Table 2:** Knowledge of officers in charge of primary healthcare facilities on *T. solium* tapeworm and *T. solium* cysticerci.

Variable	Participants
Proportions of OICs with respect to:	
Awareness to *T. solium* adult worms	133 (87.5)
Awareness to *T. solium* cysticerci in pigs	72 (47.5)

Proportions of respondents with ≥50% knowledge scores	
OICs with ≥50% scores regarding *T. solium* tapeworm knowledge	118 (77.8)
OICs with ≥50% scores regarding *T. solium* cysticerci knowledge	64 (42.1)

Proportions of respondents with<50% knowledge scores	
OICs with <50% scores regarding *T. solium* tapeworm knowledge	33 (21.7)
OICs with <50 scores regarding *T. solium* cysticerci knowledge	88 (57.9)

Knowledge on the number of hosts involved in the life cycle of the parasite	
OICs correctly mentioned the two hosts	86 (56.6)
OICs correctly mentioned one host	45 (29.6)
OICs wrongly mentioned the hosts	21 (13.8)

Knowledge on the means of human infection by *T. solium* cysticerci	
OICs correctly mentioned the means of human infection by the cysticerci	59 (38.8)
OICs mentioned eating food and drinking water contaminated by *T. solium* eggs	42 (27.6)
OICs mentioned either eating food or drinking water contaminated by *T. solium* eggs	17 (11.2)

OICs, officers in charge.

**Table 3:** Officers in charge of primary healthcare facilities based on knowledge levels regarding *T. solium* adult tapeworms and *T. solium* cysticerci.

Knowledge category	Excellent (%)	Good (%)	Average (%)	Poor (%)
Knowledge on *T. solium* tapeworm	98 (64.4)	0 (00.0)	21 (13.9)	33 (21.7)
Knowledge on *T. solium* cysticerci	42 (27.6)	22 (14.5)	0 (00.0)	88 (57.9)

**Table 4:** Knowledge of meat inspectors regarding *T. solium* adult tapeworm and *T. solium* cysticercosis diagnosis.

Variable	Number (*n* = 108) (%)
Proportions of MIs with respect to:	
Presence of slaughter slabs in their localities	10 (9.3)
AHP training background	44 (40.7)
Awareness to *T. solium* adult worms	88 (81.5)
Awareness to *T. solium* cysticerci in pigs	107 (99.1)

Proportions of respondents with ≥50% knowledge scores	
MIs with ≥50% scores regarding *T. solium* tapeworm knowledge	46 (42.6)
MIs with ≥50% scores regarding *T. solium* cysticerci knowledge	88 (81.2)
MIs with ≥50% scores regarding risk factors for *T. solium* tapeworm lifecycle maintenance	71 (65.7)
MIs with ≥ 50% scores regarding *T. solium* cysticercosis risk-based meat inspection	39 (36.1)

Decisions regarding *T. solium* cysticercosis-positive pork	
Total condemnation and burying	44 (41.2)
Total condemnation and burning	26 (24.0)
Passing conditional to thorough cooking	7 (6.6)
Passed the carcass if the cysticerci were less than 5	2 (1.6)
Passed the carcass if the cysticerci were less than 12	1 (1.1)

Targeted organs for diagnosing porcine cysticercosis	
Tongue	19 (17.6)
Masseter muscles	16 (15.1)
Leg muscles	15 (14.2)
Heart muscles	15 (13.6)
Psoas muscles	12 (10.9)
Neck muscles	10 (8.9)
Ribs muscles	9 (8.7)
Liver	6 (5.3)
Abdominal muscles	4 (3.5)
Bladder, intestines, and stomach	2 (2.2)

AHP, animal health profession; MIs, meat inspectors.

**Table 5:** Meat inspectors' knowledge on *T. solium* cysticercosis/taeniosis and porcine cysticercosis risk-based pork carcass inspection.

Knowledge category^*∗*^	Excellent (%)	Good (%)	Average (%)	Poor (%)
Knowledge about *T. solium* tapeworms	26 (24.1)	0 (00.0)	20 (18.5)	62 (57.4)
Knowledge regarding *T. solium* cysticerci	39 (36.1)	17 (15.7)	49 (45.4)	3 (02.8)
Knowledge on risk factors for tapeworm lifecycle maintenance	54 (50.0)	17 (16.0)	0 (00.0)	37 (34.0)
Knowledge on porcine cysticercosis risk-based meat inspection	4 (03.7)	10 (09.3)	25 (23.1)	69 (63.9)

^*∗*^Graded by converting the correct responses into percentage and then grouping the percentage scores into four as follows: excellent knowledge (those who scored 70% or more), good knowledge (scored between 60% and 69%), average knowledge (those who scored between 50 and 59%), and poor knowledge (those who scored below 50% of the total scores).

**Table 6:** Distribution by geographical and administration levels of study respondents.

Factor	Number of respondents (*n* = 33)
Sex (*n* = 33)	
Male	28
Female	5

National (*n* = 2)	
Ministry of Livestock and Fisheries Development	1
Ministry of Health	1

Region (*n* = 14)	
Manyara	3
Dodoma	5
Ruvuma	3
Iringa	2
Arusha	1

Districts (*n* = 17)	
Babati	4
Mbulu	5
Kongwa	2
Mbinga	4
Nyasa	2

Profession (*n* = 33)	
Medical doctor	15
Nurse	1
Veterinary doctor	10
Animal scientist	5
Veterinary paraprofessional	2

Work station (*n* = 33)	
District hospital	8
Referral hospital	6
Zonal Veterinary Investigation Centre (ZVC)	3
Tanzania Veterinary Laboratory Agency (TVLA)	2
District Livestock and Fisheries Department	8
Regional Administrative Secretariat	4
Ministry of Livestock and Fisheries Development	1
Ministry of Health	1

Sector (*n* = 33)	
Medical	16
Livestock	17

Level of medical healthcare facility (*n* = 14)	
District hospital	8
Referral hospital	6

## Data Availability

The datasets used and/or analyzed during the current study are available from the corresponding author upon request.

## Abbreviations

LMICs: Low and middle-income countries
TSCT: *Taenia solium* cysticercosis/taeniosis
OICs: Officers in charge
PHFs: Primary healthcare facilities
MIs: Meat inspectors
NCC: Neurocysticercosis
PCC: Porcine cysticercosis
CT: Computed tomography
MRI: Magnetic resonance imaging
LAMP: Loop-mediated isothermal amplification
BESS: Base excision sequence scanning
CSF: Cerebrospinal fluid
FFPE: Formalin-fixed paraffin-embedded
WCA: Whole crude *T. solium* cysticercus antigen
DNA: Deoxyribonucleic acid
EITB: Enzyme-linked immunoelectrotransfer blot
ZVCs: Zonal veterinary investigation centres
TVLA: Tanzania Veterinary Laboratory Agency
DMD: District Medical Department
DLFDD: District Livestock and Fisheries Development Department
MDs: Medical doctors
DVO: District veterinary officer
DLFO: District livestock and fisheries officer
CMHP: Clinical medical health profession
OMPs: Other medical professionals
AHP: Animal health profession
WHO: World Health Organization
OIE: International Organisation for Animal Health
FAO: Food and Agricultural Organization.

## Supplementary Materials

Supplementary Materials Supplementary material contains structured questionnaires for quantitative data collection from meat inspectors and officers in charge of primary healthcare facilities. It contains two different questionnaires for two categories of respondents, namely, meat inspectors and officers in charge of primary healthcare facilities.
