# Supplementary material for: Challenges in the Diagnosis of Taenia solium Cysticercosis and Taeniosis in Medical and Veterinary Settings in Selected Regions of Tanzania: A Cross-Sectional Study
Source: Vet Med Int. 2022 Jun 30;2022:7472051. doi: 10.1155/2022/7472051 (PMC9262556; doi:10.1155/2022/7472051)
Supplement: Supplementary Materials — Supplementary material contains structured questionnaires for quantitative data collection from meat inspectors and officers in charge of primary healthcare facilities. It contains two different questionnaires for two categories of respondents, namely, meat inspectors and officers in charge of primary healthcare facilities. [file 7472051.f1.docx]

**Supplement (S_1)_**

1. **QUESTIONNAIRE INTERVIEW GUIDE (QUANTITATIVE DATA COLLECTION)**
2. **MEAT INSPECTORS**

Dear respondent, we thank you for your willingness to spare your time to respond to questions in this questionnaire. Please respond to this questionnaire truthfully. All responses will be kept confidential and will not be used against you in any way. The information provided will be used for research purposes only.

**Name: ………………….....Sex: ……..Telephone No:……………… Region:…………**

**District:………………….Ward:………………Village:…………………………………..Date of Interview:………………GPS co-ordinates: Longitude N ______latitude E __________**

1. Highest education level. **(write the latter of the answer selected by the respondent inside the brackets provided)**
   1. Certificate in General Agriculture
   2. Certificate in Agricultural Mechanization
   3. Diploma in Agricultural Mechanization
   4. Diploma in General Agriculture
   5. Certificate in Animal Production
   6. Certificate in Range and Pasture Management
   7. Diploma in Animal Production
   8. Certificate in Animal Health
   9. Certificate in Animal Health and Production
   10. Diploma in Animal Health
   11. Diploma in Animal Health and Production
   12. Other course ( )
2. How long has it passed since graduation at your professional education?.....months
3. Do you know a worm called *Taenea solium?* **(Write the latter of the answer selected by the respondent inside the brackets provided).**
   1. Yes
   2. No ( )

**If his/her response to question 4 is ‘Yes’ continue with question 5 otherwise skip to**

**question 8.**

1. You are provided with a list of answers that describe *T. solium* tapeworm. Select the answers that you think they best describe the tapeworm. (Put a tick **(√) beside the letter of the answer selected by the respondent).**
2. A thread like round worm residing in the stomach of pig
3. It is more than 2 metres long
4. A pigmented tapeworm with scolex (head)
5. A 3 to 4 millimetres long flatworm
6. It is made up of four suckers each with double rows of hooks with which it attaches on the walls of the intestine.
7. Residing in the liver of human , carrying eggs in it
8. Has proglottids which contain eggs
9. It causes cyst in various organs of pigs, goats and sheep

1. How many hosts does a tapeworm have? **(Write the latter of the answer selected by the respondent inside the brackets provided).**
   1. One
   2. Three
   3. Two

(d) Five ( )

1. **Write the latter of the answer selected by the respondent inside the brackets provided,**

The mentioned host(s) in question 5 is (are);

- 1. Dog
  2. Dog and Pig
  3. Cattle and Dog
  4. Pig and Human
  5. Human and Cattle
  6. Pig and Dog
  7. Cattle ( )

1. Do you know *T. solium* cyst? (**Write the latter of the answer selected by the respondent inside the brackets provided)**
   1. Yes
   2. No ( )

**If his/her response to question 8 is ‘Yes’ continue with question 9 otherwise skip to**

**question 14**

1. You are provided with a list of statements that describe *T. solium* cyst, you are required to choose the statements that you think they best describe the cysticercus. **(Put a tick (√) beside the letter of the answer selected by the respondent).**

A *T. solium* cyst is

1. A fluid filled sac
2. About 0.5 to 2cm in diameter
3. A white –greyish coloured sac
4. A blood filled sac-like membrane
5. About 2 to 10mm water filled sac
6. Resides in the brain
7. Resides in many tissues and organ including brain
8. A pus filled sac in the tissues of an infected man and pigs

1. Which of the following statements is true about *T. solium* cyst contribution into the life cycle of the tapeworm**? (Write the latter of the answer selected by the respondent inside the brackets provided).**
2. Is a larval stage of the tapeworm ingested by human through improperly cooked infected pork in which it develops into an adult *T. solium* tapeworm
3. Contains eggs which develop into an adult *T. solium* tapeworm
4. It is ingested by scavenging dogs and cats in which they develop into an adult tapeworm. ( )
5. Choose the risk factors which account for *T. solium* adult and larval stages of the parasite infection and life circle maintenance. **(Put a tick (√) beside the letter of the answer selected by the respondent).**
6. Improper handling of cysticerci infected pork
7. Unhygienic preparation of food
8. Keeping dogs and pigs together
9. Improper use of latrines
10. Feeding dogs with cysticerci infected pork
11. Drinking of unsafe water which may be contaminated by eggs of *T. solium* tapeworm
12. Eating bush meat
13. Stigmatization of epileptic patient
14. Negligence to treatment of infected *T. solium* tapeworm carriers

1. **Write the latter of the answer selected by the respondent inside the brackets provided.**

A pig gets infected by *T. solium* cysticercosis through:

- 1. Scavenging on human faeces or drinking on water and eating feeds contaminated by *T. solium* eggs
  2. Eating on *T. solium* cysticerci infected pork
  3. I don’t know ( )

1. Choose from the list below the way(s) by which human being gets infected by *T. solium*

Cysticorcis. **(Put a tick (√) beside the letter of the answer selected by the respondent).**

- 1. Eating improperly cooked *T. solium* cyst infected pork
  2. Eating food contaminated by *T. solium* eggs
  3. Drinking water contaminated *by T. solium* eggs
  4. Getting bitten by infective tick and mosquito
  5. Other……………….
  6. I don’t know

1. Have you ever attended any refresher short course training on meat inspection? **(Write the latter of the answer selected by the respondent inside the brackets provided)**
   1. Yes
   2. No ( )

**If the response to question 14 is ‘Yes’ proceed with question 15 otherwise skip to**

**question 16**

1. How long has it passed since you last attended the course? ……………months
2. Do you have pig slaughter slabs in your working area? **(write the latter of the answer selected by the respondent inside the brackets provided)**
3. Yes
4. No ( )

**If the response to question 16 is ‘Yes’ proceed with question 17 otherwise skip to**

**question 18**

1. How many pig slaughter slabs/houses do you have in your working area?....
2. Are you facilitated with any means of transport to help you move around doing meat

inspection?

1. Yes
2. No ( )

1. Do you know the organ/areas to concentrate the most if you want to locate *Cysticercus*

*sellulosae?* **(Write the latter of the answer selected by the respondent inside the brackets provided).**

1. Yes
2. No ( )

1. If your answer in question 19 above is ‘Yes’, choose from the list below the most *T. solium* cysticerci predilection sites in pigs. **(Put a tick (√) beside the letter of the answer selected by the respondent).**
2. Tongue
3. Heart muscles
4. Rib muscles
5. Psoas major and minor
6. Masseter muscle
7. Neck
8. Legs
9. Abdominal muscles
10. Liver
11. Bladder, intestines and stomach
12. Other…………
13. Explain briefly how do you go about doing meat inspection in pigs …………………….

1. What do you do with a *T. solium* cysticerci infected pork carcass? **(Put a tick (√) beside the letter of the answer selected by the respondent).**
2. Burry the carcass
3. Demand them to burry or burn only part of the affected carcass
4. Burn the carcass
5. Pass for human consumption under the condition that it should be thoroughly cooked
6. If the carcass owner keeps dog I allow him to take and feed the carcass to dogs
7. Because pork owners are always arrogant I allow them to take the carcass but advice not to sale in their butchers
8. I usually don’t know what to do
9. Other

**THANK YOU FOR ACCEPTING TO PARTICIPATE IN THIS INTERVIEW**

1. **HEALTH WORKERS**

**Introduction**

Dear respondent, we thank you for your willingness to spare your time to respond to questions in this questionnaire. Please respond to this questionnaire truthfully. All responses will be kept confidential and will not be used against you in any way. The information provided will be used for research purposes only.

**Name: ………………….....Sex: ……..Telephone No:……………… Region:…………**

**District:………………….Ward:………………Village:…………………………………..Date of Interview:………………GPS co-ordinates: Longitude N ______latitude E**

1. Which of the following is your professional background**? (Write the latter of the answer selected by the respondent inside the brackets provided)**
2. Medical Doctor
3. Assistant Medical Doctor
4. Clinical Officer
5. Assistant Clinical Officer
6. Other…………… ( )
7. For how long have you been working since you first graduated with your current profession?......months
8. Do you know *Taenea solium* tapeworm? **(Write the latter of the answer selected by the respondent inside the brackets provided)**
   - 1. Yes
     2. No ( )

**If the response to question 3 is ‘Yes’ continue to question 4, otherwise skip to question 7**

1. You are provided with a list of answers that describe *T. solium* tapeworm. Select the answers that you think they best describe the tapeworm. **(Put a tick (√) beside the letter of the answer selected by the respondent).**
2. A thread like round worm residing in the stomach of pig
3. It is more than 2 metres long
4. A pigmented tapeworm with scolex (head)
5. A 3 to 4 millimetres long flatworm
6. It is made up of four suckers each with double rows of hooks with which it attaches on the walls of the intestine.
7. Residing in the liver of human , carrying eggs in it
8. Has proglottids which contain eggs
9. It causes cyst in various organs of pigs, goats and sheep
10. How many hosts are involved in the tapeworm life cycle? **(Write the latter of the answer selected by the respondent inside the brackets provided)**
    1. One
    2. Three
    3. Two
    4. Five ( )
11. Mentioned hosts in question 5 above are… **(Write the latter of the answer selected by the respondent inside the brackets provided)**
    1. Dog
    2. Dog and Pig
    3. Cattle and Dog
    4. Pig and Human
    5. Human and Cattle
    6. Pig and Dog
    7. Cattle ( )
12. Do you have a laboratory with capacity to diagnose heminthosis in your healthcare facility? **(Write the latter of the answer selected by the respondent inside the brackets provided)**
13. Yes
14. No ( )

**If the response to this question is ‘Yes’ proceed to question 8 otherwise skip to**

**question 10**

1. If your answer in question 7 above is ‘Yes’, which sample do you collect for *T. solium* diagnosis? **(Put a tick (√) beside the letter of the answer selected by the respondent).**
   - 1. Blood
     2. Stool
     3. Urine
     4. Blood and stool
     5. Blood and urine
     6. Stool and urine
     7. I don’t know
     8. I don’t collect any sample
2. How do you diagnose *T. solium* tapeworm parasite at your health facility. **(Put a tick (√) beside the letter of the answer selected by the respondent).**
   1. Checking for the parasite eggs in the stool
   2. Checking for the parasite ovaries arrangement in the proglottid in the stool
   3. Checking for the parasite scolex in the stool
   4. Antibodies/antigens in serum sample
3. How do you diagnose *T. solium* tapeworm parasite at your healthcare facility. **(Put a tick (√) beside the letter of the answer selected by the respondent).**
4. Through explanation by the patient or child’s mother that she has seen

pieces of tapeworm in the stool

1. I don’t know
2. We don’t diagnose it here
3. Other…..
4. Do you know Cysiticercus sellulosae? **(Write the latter of the answer selected by the respondent inside the brackets provided)**
   - 1. Yes
     2. No ( )

**If the response to this question is ‘Yes’ proceed to question 12, otherwise skip to**

**question 15**

1. You are provided with a list of statements that describe *T. solium* cyst. You are required to choose the statement (s) that best describes the parasite. A *T. solium* cyst is; **(Put a tick (√) beside the letter of the answer selected by the respondent).**
2. A fluid filled sac
3. 0.5 to 2cm in diameter
4. A white –greyish coloured sac
5. A blood filled sac-like membrane
6. About 2 to 10mm water filled sac
7. Resides in the brain
8. Resides in many tissues and organ including brain
9. A pus filled sac in the tissues of an infected man and pigs

1. How does human get infected with the *Cysticercus sellulosae.* **(Write the latter of the answer selected by the respondent inside the brackets provided)**
   - 1. Eating pork infected with *T. solium* cyst
     2. Eating food or drinking water contaminated with *T. solium* eggs
     3. Both a and b are correct
     4. I don’t know ( )

1. Which of the following statements is true about *T. solium* cyst contribution into the life cycle of the tapeworm? **(Write the latter of the answer selected by the respondent inside the brackets provided)**
2. Is a larval stage of the tapeworm ingested by human through improperly cooked infected pork in which it develops into an adult *T. solium* tapeworm
3. Contains eggs which develop into an adult *T. solium* tapeworm
4. It is ingested by scavenging dogs and cats in which they develop into an adult tapeworm. ( )
5. Choose from the following list, the risk factor(s) which account for *T. solium* adult and larval stages of the parasite infection and life circle maintenance. **(Put a tick (√) beside the letter of the answer selected by the respondent).**
6. Improper handling of cysticerci infected pork
7. Unhygienic preparation of food
8. Keeping dogs and pigs together
9. Improper use of latrines
10. Feeding dogs with cysticerci infected pork
11. Drinking of unsafe water which may be contaminated by eggs of *T. solium*

tapeworm

1. Eating bush meat
2. Stigmatization of epileptic patient
3. Negligence to treatment of infected patient
4. Are you aware of a medical condition called human neurocysticercosis? **(Write the latter of the answer selected by the respondent inside the brackets provided)**
   - 1. Yes
     2. No ( )
5. If your answer in 16 above is ‘Yes’, what is neurocysticercosis? **(Write the latter of the answer selected by the respondent inside the brackets provided)**
   - 1. Presence of cysticerci in the central nervous system
     2. Destruction of the general performance of the central nervous system
     3. Presence of a foreign body in the brain
     4. Presence of *T. solium* tapeworm in the central nervous system
     5. I don’t know ( )

**THANK YOU FOR ACCEPTING TO PARTICIPATE IN THIS INTERVIEW**
